# Effectiveness of Triple Benefit Health Education Intervention on Knowledge, Attitude and Food Security towards Malnutrition among Adolescent Girls in Borno State, Nigeria

**DOI:** 10.3390/foods11010130

**Published:** 2022-01-05

**Authors:** Ruth Charles Shapu, Suriani Ismail, Poh Ying Lim, Norliza Ahmad, Hussaini Garba, Ibrahim Abubakar Njodi

**Affiliations:** 1 Department of Community Health, Faculty of Medicine and Health Science, University Putra Malaysia, Serdang 43400, Selangor, Malaysia; ruthyshapu52@gmail.com (R.C.S.); pohying_my@upm.edu.my (P.Y.L.); lizaahmad@upm.edu.my (N.A.); 2 College of Nursing and Midwifery, Damboa Road, Maiduguri 600252, Borno State, Nigeria; 3 Department of Physical and Health Education, University of Maiduguri, Maiduguri 600230, Borno State, Nigeria; garbahussaini1759@gmail.com (H.G.); ibrahimnjodi@gmail.com (I.A.N.)

**Keywords:** triple benefit health education intervention, adolescent girls, knowledge, attitude, food security, KoBo Collect Toolbox, generalized estimating equation

## Abstract

Knowledge and attitude are essential components of food security as malnutrition remains a critical public health concern among adolescents. The study evaluates the effectiveness of a Triple Benefit Health Education Intervention on knowledge, attitude and food security towards malnutrition among adolescent girls. This was a cluster randomized controlled trial among 417 randomly selected adolescent girls aged 10 to 19 years old in Maiduguri, Borno state, Nigeria from October 2019 to March 2020. About 208 respondents were assigned to experimental while 209 to control group, respectively, using an opaque sealed envelope. A structured questionnaire using KoBo Collect Toolbox was used for the collection of data at baseline, three and six-months post intervention while the data collected were analyzed using generalized estimating equation (GEE). The outcome of the baseline shows no statistically significant difference in sociodemographic characteristics, knowledge, attitude and food security between experimental and control groups. The study reveals a statistically significant difference between experimental and control groups for knowledge (*p* < 0.001; *p* < 0.001), attitude (*p* < 0.001; *p* < 0.001) and food security (*p* = 0.026; *p* = 0.001) at three and six-months post intervention, respectively. The triple benefit health education intervention package employed in this study can serve as an intervention tool to combat malnutrition among adolescent girls in Nigeria at large.

## 1. Introduction

Adolescents are the nation’s future, so there is a need for them to have an adequate knowledge of, and attitude towards, the importance of balanced diet and the importance of food security in terms of its availability, accessibility, affordability and utilization, as it forms the basis for a healthy life, mental development, intellectual abilities, physical growth and strength to cope with daily activities [1,2]. The effect of malnutrition and food security among adolescents, and especially during their childhood years, is apparent. Food insecurity at the adolescent stage is the possible cause of numerous chronic diseases during adult life. Preventive effort to minimize both short and long term consequences of malnutrition should start at the early childhood stage, all through adolescence and even beyond, to the benefit of health and later life [3,4,5]. The adverse effect of malnutrition with an increasing trend among adolescents globally reflect poor knowledge, attitude and insufficient food intake [6]. The various forms of malnutrition are persistent and co-exist within various families, communities, regions and countries of the world. Nearly one third of the world population are facing one or more forms of malnutrition, and with this rising trend malnutrition may rise to one half by 2030, rendering the objective of ending malnutrition by 2030 unachievable. The negative health effect of malnutrition, the risk of premature death and the prevalence of non-communicable diseases is increasingly becoming unbearable in middle and low income countries [7,8,9]. 

Globally, it has been reported that many children and adolescents (young people) are not able to develop and grow to reach their full potential and become productive in life due to an inadequate diet to meet their daily nutritional needs. In order for children and adolescents to have good nutritional intake, it is important for them, especially adolescent girls, to have a nutritious, affordable, safe, and sustainable diet to meet their everyday needs. The triple burden of malnutrition (undernutrition, over nutrition and micronutrient deficiency) has become a threat to the growth, development and survival of children and adolescents, further endangering the productivity of nations, as a result of the poor quality and quantity of diet consumed [10]. Knowledge and attitude are significant components in the transformation of food security especially among children and adolescents who will have to be accountable for the implementation of most of the nutrition related policies as they advance towards their maturity. Healthy and sufficient nutrition is indispensable for children and adolescents at all stages of their growth and development, from playing to learning and being happy [11]. Findings from a study on high-return investments in school health regarding increased participation and learning suggests that the first 1000 days is important in a child’s life, but not sufficient for the survival of the child. Investment in the 8000 days of childhood through adolescence requires intense intervention in three possible stages. Phase one (5 to 9 years) is when mortality rate is higher due to malnutrition and infection, thereby restraining growth and development, referred to as middle childhood growth and consolidation stage. Phase two (10–14 years) is the growth spurt stage when adolescents require an adequate and balanced diet for their healthy growth and development. Phase three (15 to 19 years), also called the growth and consolidation stage, supports the maturation of the brain, emotional control and socialization. The suggested 8000 days will enable a catch up from growth failure at the early stage [12,13,14]. Poor diet as a result of food insecurity, poor knowledge and attitude are the major underlying causes of malnutrition, with high economic costs, placing the burden of intergenerational malnutrition across generations to come in the future [7]. 

Health education intervention for adolescents in a school setting provides them with the required skills, knowledge and attitudes for healthy selection and utilization of food. Unhealthy selection of food, inadequate information on the importance of food and food groups, malnutrition and its consequences and lack of a kitchen garden among adolescents can increase the burden of malnutrition, which can be mitigated through health education intervention. Previous studies have revealed that health education intervention lays a foundation for the improvement of nutritional knowledge and attitudes relevant to healthy living [1,15,16,17]. Addressing the root causes of malnutrition requires a common understanding of the problems and the possible solutions therein, health education intervention across all age groups, both in school and out of school, and the political will in terms of governance, implementation and alignment of policies [8]. The rising trend of poverty and low income has remained a complex, chronic and pervasive problem; an estimated 40.1% of the Nigerian population live below the poverty line where children from the poorest economic quartile were reported to be four times more likely to be malnourished compared with children from the richest households. Malnutrition is a serious consequence of food insecurity [18,19]. The level and dimension of hunger and food insecurity have become a public health concern in Nigeria. Agricultural production in Nigeria is largely dependent on rainfall at a subsistent level on a small scale. Government investment in agricultural production has not contributed to the reduction of malnutrition significantly to meet the national development goal, as the inadequate storage system, crop seasonality, and inadequate transport system has significantly influenced food distribution in Nigeria [18,20,21]. Persistent humanitarian crises especially in the north-eastern part of Nigeria have greatly exposed the people to untold hardship. 

Since there are high burdens of malnutrition among children and adolescents that may heighten health related problems, there is need for nutrition sensitive intervention, especially in low and middle-income countries, which must be addressed as a priority. The sustainable development goals have pointed out ways of reducing the determinant of malnutrition, including no poverty, no hunger, quality education, and gender equality, but for the adolescent age group social determinants and nutrition sensitive intervention must be addressed to improve their health and wellbeing. Healthy adolescents who are protected from morbidity and early pregnancy are less likely to develop all forms of malnutrition during adolescence and adulthood, and more likely to reduce the occurrence of non-communicable diseases, have optimal maternal and birth outcomes, and enjoy increased productivity and work capacity [6]. 

There are no existing data on the overall current prevalence of malnutrition, knowledge, attitudes and food security related issues among adolescents in Nigeria. The trend of malnutrition among women aged 15–49 years, with adolescents included, has been stable for the past 10 years, reporting 12% from 2008 to 2018 [22]. Furthermore, the prevalence of acute malnutrition among older adolescents was 19% and about four times higher compared to 4% among adult women aged 20–49 years in Nigeria. About 6.1% out of 14% of women between 15–49 years pregnant particularly in the northeast and northwest Nigeria were adolescent girls aged 15–19 years old. More so, anemia is also a trending concern among 58% of women aged 15–49 years leading to increased burden of maternal mortality, poor birth outcome and reduced productivity [22,23]. Childbearing among adolescent girls is significantly associated with a high risk of pregnancy complication outcome [24]. The findings from the 2018 national survey call for urgency in the development of an intervention to improve nutrition related knowledge and attitude among adolescent girls for better health, birth outcome and nutrition throughout their life cycle. Taming malnutrition among adolescent girls is key to improving the nutritional status of the family and the entire population.

There are several policies and programs put in place by the Nigerian government to address the problem of malnutrition among children including the national policy on food and nutrition in Nigeria [25,26], the national strategic plan of action (health sector response), the food security bill, the National Plan of Action on Food and Nutrition in Nigeria and the micronutrients control program, among others, to address the issue of malnutrition and food insecurity at all levels in Nigeria [27]. Nevertheless, the implementation of these programs and policies continues to be a challenge, with a persistently high level of malnutrition among children [27,28,29]. There are no existing nutrition programs and policies for adolescents as the national food and nutrition policy in Nigeria made little or no reference to nutrition-related issues regarding this important specific population (adolescents). The strategic plan of action and most of the intervention target children that are below the age of five years, pregnant and lactating women, failing to notice the plight of adolescents. There is a need to revisit the national strategic plan of action in Nigeria to include interventions targeting adolescents for the good of the future, in all its seriousness. Nonetheless, amid the trending burden of malnutrition, there is no existing comprehensive health education program on malnutrition targeting adolescents in Nigeria; though there are policies to reduce the burden of malnutrition, the implementation is still a challenge. The triple benefit health education intervention was introduced to look beyond the 1000 days of the child’s life (from conception to the second birthday), before the preconception period to productive adult life in the future, and the health and wellbeing of their offspring.

Usually, researchers incorporate a theory-based approach for their research to be successful. The information, motivation, behavioral skills model (IMB) has been used in promoting nutrition-related practices and the development of preventive intervention [30]. Although the IMB model works at the individual level, the construct of the theory is useful in presenting health behavioral changes through making individuals well informed by using their attitude and perception as motivation to make them comply and act, thus possessing the essential behavioral skills for effective achievement. The information, motivation, behavioral skills model was used in developing the Triple Benefit Health Education Intervention to improve knowledge, attitude, and food security among adolescent girls. The study is named Triple Benefit because it will improve the health of adolescent girls now, improve their productivity and well-being when she fully-grown in the future and minimize health threats and improve nutritional status and wellbeing of future offspring. Knowledge, attitude and food security towards malnutrition and nutrition-related studies among adolescents have not yet been studied in the north-eastern part of Nigeria comprising Borno, Yobe, Adamawa, Bauchi, Gombe and Taraba states from the 36 states in Nigeria. The study tends to look at what is the effectiveness of the Triple Benefit Health Education Intervention on knowledge, attitude, and food security regarding malnutrition among adolescent girls in Maiduguri Metropolitan Council, Borno State, Nigeria.

## 2. Materials and Methods

### 2.1. Study Design and Population 

A school-based single blinded cluster randomized controlled trial designed was used to evaluate the effectiveness of the triple benefit health education intervention among adolescent girls in four randomly selected secondary schools in Maiduguri, Borno state, Nigeria. A two population proportion formula was used to calculate the population size [31], based on previous study [32], considering effect size = 1.3, and non-response rate of 20%, giving a total population size of 424.

### 2.2. Inclusion and Exclusion Conditions 

Inclusion criteria were government secondary schools, schools with full secondary school, schools with boys and girls or girls school only, schools within Maiduguri only and adolescent girls between 10 and 19 years old. Schools not owned by the government, primary schools and schools with women and not girls were excluded. 

### 2.3. Duration of Study and Recruitment of Participants

This study was conducted between October 2019 and March 2020. A two-stage sampling method was adopted to randomly select four schools using a software number generator, and a total of 424 were randomly selected from six arms of the schools (JSS1 to SS3) to partake in the study. About 212 participants were recruited into the experimental group and 212 into the control group; each group was allocated using the simple random method, the allocation was performed as a cluster into the experimental and control groups using opaque sealed envelopes. However, 417 were eligible for participation in the study. Seven participants were excluded from the study, of which three were more than 19 years old, while parents of the four students did not give their consent. About 417 respondents brought back the signed consent form. Participants were interviewed using KoBo Toolbox at baseline with 208 in the intervention group and 209 in the control group, respectively.

### 2.4. Intervention Stratebt

The triple benefit health education intervention module was guided by the information, motivation, behavioral skill (IMB) theory to educate adolescent girls on knowledge, attitudes and food security. The experimental group in this study received the triple benefit health education intervention twice in a month for three months. The module was developed with the clear intention of reducing the burden of malnutrition and ultimately improving the health, well-being and the health of the future offspring of adolescent girls. The Triple Benefit Health Education Intervention module was subdivided into six modules and was conducted for three months with the experimental group by the facilitator. Each session lasted for 1 h:30 min, and the topics covered in the sessions are presented in Table 1. The control group received education twice a month for three months on malaria prevention.

### 2.5. Study Instruments and Variables 

A questionnaire was used for the collection of data, using KoBo Toolbox, on sociodemographic characteristics, knowledge, attitude and food security. The questionnaire was developed by the researcher in English.

#### 2.5.1. Demographic Features

This segment comprises 15 questions on sociodemographic characteristics including age, ethnicity, class in school, religion, household size, place of residence, household income, head of household, age of father, father’s education, father’s occupation, age of mother, mother’s education, mother’s occupation, and family type. This section has both nominal and continuous scale variables.

#### 2.5.2. Knowledge, Attitude and Food Security

The knowledge questionnaire was adapted from the Food and Agricultural Organization of the United Nations [33] and consists of 28 questions with yes, no, or don’t know as options; yes responses were scored one while no or don’t know were scored zero. Total score for knowledge was 28; scores less than 50% were considered as poor knowledge, while scores greater or equal to 50% were considered good knowledge [34].

In this study the attitude questionnaire was adapted from the Food and Agricultural Organization of the United Nations [33]. Attitude questions consist of 17 statements on a five point scale from strongly disagree to strongly agree (1 to 5). The total attitude score was 85; scores less than the mean score were considered poor attitude while scores equal or greater than the mean were considered as showing good attitude [35].

Food security questions were adapted from those for older children [36] and consist of nine statements with options as never, sometimes and a lot, with scores of 1, 2 and 3, respectively; never was recoded as ‘0’, sometimes and a lot were recorded as ‘1’. Food security has a total score of nine; 0 to 1 were considered food secure, 2 to 5 low food secured and 6 to 9 very low food security, respectively [37]. 

### 2.6. Data Collection and Analysis

Data were collected at baseline, three and six-month post intervention using KoBo Toolbox and were analyzed using SPSS version 25. Variables that are continuous were presented as mean and standard deviation before being categorized, while categorical variables were presented in the form of frequency and percentage. The differences between experimental and control group for each demographic feature were determined using Chi-square at baseline, whereas for differences in overall scores between experimental and control group for knowledge, attitude and food security at baseline, independent *t*-test was used. A generalized estimating equation was used to evaluate the changes between experimental and control group at three and six-months post intervention. Statistical significance was determined using *p*-value < 0.05 for all the comparisons in this study. 

### 2.7. Ethics 

Approval for ethics was obtained from Universiti Putra Malaysia Ethical Committee UPM/TNCPI/RMC/JKEUPM/1.4.18.2 (JKEUPM). Permission was obtained from the Ministry of Education. Written consent from parent/guardian and respondents was obtained before the intervention. The study was also registered with Pan African Clinical Trials Registry (PACTR201905528313816). 

## 3. Results

This study evaluates the effectiveness of a triple benefit health education intervention in improving knowledge, attitude and food security among adolescent girls. Out of a total of 424 participants, 417 gave their consent to partake in the study. A total of 208 were assigned to the experimental group, while 209 to the control group. There was a response rate of 100% at baseline, 98.1% at three-months and 96.6% at six-months post-intervention in both groups, respectively. Intention to treat analysis was employed and all randomized participants were included in the analysis by the imputation of the mean of the responses at three and six-months post-intervention [38]. The proportion of missing data due to attrition was 4% in the experimental group and 2% in the control group. 

### 3.1. Baseline Results

#### 3.1.1. Sociodemographic Characteristics at Baseline

Table 2 reveals the baseline demographic features of respondents in the experimental and control groups. There was no statistically significant difference in socio-demographic characteristics between experimental and control groups at baseline.

#### 3.1.2. Knowledge, Attitude and Food Security at Baseline

Table 3 indicates that the mean ± SD for knowledge score between experimental and control groups at baseline was 8.87 ± 4.3. The scores range from 0 to 19. There was no statistically significant difference between experimental and control group at baseline for knowledge (*p* = 0.589). The mean ± SD for attitude score between experimental and control group at baseline was 53.36 ± 5.0. The scores range from 41 to 70. There was no statistically significant difference between experimental and control group for attitude at baseline for attitude (*p* = 0.744). The mean ± SD for food security score between experimental and control group at baseline was 5.49 ± 3.5. The scores range from 0 to 9. There was no statistically significant difference between experimental and control groups for food security at baseline (*p* = 904).

### 3.2. Effectiveness of Triple Benefit Health Education Intervention on Knowledge, Attitude and Food Security among Respondents

The participants in the experimental and control groups were compared on knowledge, attitude and food security towards malnutrition at three and six-month post intervention using generalized estimating equation (GEE). The following are the results of the changes in knowledge, attitude and food security at 3 and 6-month post-intervention.

#### 3.2.1. Comparison of Knowledge between Experimental and Control Groups and Time Points (Baseline, 3 and 6-Months Post-Intervention) Respectively

Table 4 shows that participants in the experimental group have higher odds of having good knowledge compared to control group (AOR = 6.380, 95% CI: 4.665–8.725, *p* < 0.001). Participants at 3 and 6-month post-intervention have higher odds of having good knowledge compared to participants at baseline respectively (AOR = 9.595, 95% CI: 6.371–14.449, *p* < 0.001; AOR = 14.993, 95% CI: 9.919–22.662, *p* < 0.001). 

GEE was used to assess the effectiveness of the Triple Benefit Health Education Intervention on knowledge from baseline to three and six-months post-intervention adjusted with covariates. Factors with *p* < 0.25 at a univariate level were tested as the covariates in the final model. Table 5 shows the knowledge of participants between experimental and control groups from baseline to three and six-month post-intervention adjusted with covariates. There was no significant difference between experimental and control group for knowledge (AOR = 1.063, 95% CI: 0.594–1.901, *p* = 0.837). Participants at three and six-months post-intervention have higher odds of having good knowledge compared to participants at baseline respectively (AOR = 4.164, 95% CI: 2.321–7.471, *p* < 0.001; AOR = 5.805, 95% CI: 3.204–10.515, *p* < 0.001). 

There was a significant interaction at three and six-month post-intervention; participants at three and six-month post-intervention have higher odds of having good knowledge compared to control group at baseline, respectively (AOR = 4.407, 95% CI: 1.964–9.893, *p* < 0.001; AOR = 9.397, 95% CI: 3.915–22.471, *p* < 0.001). 

Participants in SS1 and SS2 have higher odds of having good knowledge compared to those JSS 1 (AOR = 1.797, 95% CI: 1.007–3.208, *p* = 0.047; AOR = 1.879, 95% CI: 1.135–3.109, *p* = 0.014). Participants from Chibok ethnic group have higher odds of having good knowledge compared to those from Bura ethnic group (AOR = 0.460, 95% CI: 0.228–0.927, *p* = 0.030). Participants in food secured level have higher odds of having good knowledge compared to those in very low food secured level (AOR = 1.926, 95% CI: 1.309–2.835, *p* = 0.001). Participants with good information have higher odds of having good knowledge compared to those with poor information (AOR = 2.252, 95% CI: 1.600–3.170, *p* < 0.001). Participants with good motivation have higher odds of having good knowledge compared to those with poor motivation (AOR = 1.973, 95% CI: 1.377–2.829, *p* < 0.001). Participants with good behavioral skills have higher odds of having good knowledge compared to those with poor behavioral skills (AOR = 1.466, 95% CI: 1.045–2.057, *p* = 0.027). 

#### 3.2.2. Comparison of Attitude between Experimental and Control Groups and Time Points (Baseline, Three and Six-Months Post-Intervention) Respectively

Table 6 shows that participants in the experimental group have higher odds of having good attitude compared to control group (AOR = 2.002, 95% CI: 1.619–2.476, *p* < 0.001). Participants at three and six-months post-intervention have higher odds of having good attitude compared to those at baseline respectively (AOR = 1.949, 95% CI: 1.451–2.616, *p* < 0.001; AOR = 2.276, 95% CI: 1.692–3.060, *p* < 0.001). 

GEE was used to assess the effectiveness of the Triple Benefit Health Education Intervention on the attitude of participants from baseline to three and six-months post-intervention adjusted with covariates. Factors with *p* < 0.25 at a univariate level were tested as the covariates in the final model. Table 7 shows the attitude of participants between experimental and control groups from baseline to three and six-months post-intervention, adjusted with covariates. There was no significant difference between experimental and control groups for attitude (AOR = 1.000, 95% CI: 0.674–1.482, *p* = 0.998). Participants at three-months post-intervention have higher odds of having good attitude towards malnutrition compared to those at baseline (AOR = 1.624, 95% CI: 1.807–2.428, *p* = 0.018). 

There was a significant interaction at three and six-months post-intervention; participants at three and six-months post-intervention have higher odds of having good attitude compared to control group at baseline (AOR = 1.367, 95% CI: 1.747–2.501, *p* = 0.017; AOR = 3.076, 95% CI: 1.636–5.785, *p* < 0.001), respectively. 

Islamic participants have higher odds of having good attitude compared to those from the Christian religion (AOR = 1.505, 95% CI:1.189–1.903, *p* = 0.001). Participants with normal BMI have higher odds of having good attitude compared to underweight (AOR = 1.480, 95% CI: 1.107–1.978, *p* = 0.008). Participants in low food secured level have higher odds of having good attitude compared to very low food secured level (AOR = 1.414, 95% CI: 1.031–1.939, *p* = 0.032). Participants with good information on malnutrition have higher odds of having good attitude compared to those with poor information on malnutrition (AOR = 1.645, 95% CI: 1.271–2.128, *p* < 0.001).

#### 3.2.3. Comparison of Food Security between Experimental and Control Groups and Time Points (Baseline, Three and Six-Months Post-Intervention) Respectively

Table 8 shows that participants in the experimental group have higher odds of being in food secured level compared to control group (AOR = 4.688, 95% CI: 3.654–6.015, *p* < 0.001). Participants at three and six-months post-intervention have higher odds of being in food secured level compared to those at baseline respectively (AOR = 1.356, 95% CI: 1.037–1.771, *p* = 0.026; AOR = 1.589, 95% CI: 1.223–1.064, *p* = 0.001). 

GEE was used to assess the effectiveness of the Triple Benefit Health Education Intervention on the food security level of respondents from baseline to three and six-months post-intervention adjusted with covariates. Factors with *p* < 0.25 at a univariate level were tested as the covariates in the final model. Table 9 shows the food security level of participants between experimental and control group from baseline to three and six-months post-intervention, adjusted with covariates. There was no significant difference between experimental and control groups for food security level (AOR = 0.887, 95% CI: 0.606–1.229, *p* = 0.539). There were no significant differences at three and six-months post-intervention for food security level compared to those at baseline (AOR = 0.914, 95% CI: 0.616–1.358, *p* = 0.658; AOR = 0.869, 95% CI: 0.581–1.299, *p* = 0.492), respectively. 

There was a significant interaction at three and six-months post-intervention; participants at three and six-months post-intervention have higher odds of being in food secured level compared to control group at baseline (AOR = 2.116, 95% CI: 1.197–3.740, *p* = 0.010; AOR = 2.552, 95% CI: 1.467–4.440, *p* = 0.001), respectively. 

Participants in urban areas have higher odds of being in food secured level compared to those in rural areas (AOR = 1.532, 95% CI: 1.122–2.091, *p* = 0.007). Participants whose monthly income was between ₦31,000–₦50,000 and ₦51,000 and above have higher odds of being in food secured level compared to those with monthly income less than ₦18,000 (AOR = 1.539, 95% CI: 1.099–2.153, *p* = 0.012; AOR = 2.236, 95% CI: 1.652–3.025, *p* < 0.001). Participants whose mothers’ education were primary and tertiary education have higher odds of being in food secured level compared to those whose mothers had no education (AOR = 1.739, 95% CI: 1.115–2.712, *p* = 0.015; AOR = 1.454, 95% CI: 1.044–2.025, *p* = 0.027). Participants whose sources of information were from health worker/clinic and schoolteachers have lower odds of being in food secured level compared to those whose sources of information were from social media (AOR = 0.507, 95% CI: 0.298–0.862, *p* = 0.012, AOR = 0.610, 95% CI: 0.379–0.980, *p* = 0.041). For every increase in protein, participants have higher odds of being in food secured level compared to very low food secured level (AOR = 1.003, 95% CI: 1.000–1.005, *p* = 0.032).

## 4. Discussion

Acquiring adequate nutritional knowledge and attitude will help children, adolescents and entire families to make better choices regarding foods that are convenient, available, affordable and desirable for healthy living. There is need to invest in children and adolescents globally to achieve the sustainable development goal by 2030 [10]. 

The findings in this study showed that there was no significant difference in the age of respondents between the experimental and control group at baseline, similar to studies in U.S.A and China [39,40]. Class of participants between experimental and control groups were not significant, concurring with a study in California [41]. The ethnicity of participants showed no significant difference between experimental and control group, in line with a study in Canada [42]. Religion, place of residence, household size, head of household, and education of father and mother were not significant between experimental and control, agreeing with a study in Ethiopia [43,44]. This study further revealed that there was no significant difference in age of fathers and mother, or occupation of father and mother between experimental and control, similar to a study in Iran [45]. The study overall reveals no significant differences in sociodemographic characteristics of respondents between intervention and control group at baseline, indicating that the two groups are comparable in their characteristics due to a good randomization process, Table 2.

This study hypothesized that there were no significant differences in knowledge of malnutrition between the experimental and control group at baseline; there was similar agreement with other studies in Canada, China, Los Angeles and California [32,41,42,46]. The study reveals that there was no significant difference in the attitude of participants towards malnutrition between experimental and control group at baseline. There are similarities with experimental studies conducted in Canada, South Africa, the southwestern state of Malaysia and China [3,32,42,47]. There was no significant difference in all the food security statement between the experimental and control group at baseline. Food security can be said to exist if all people at all times have cost-effective and physical access to safe, adequate and nourishing food to meet their nutritional needs for an energetic and healthy life. Globally, more than 820 million people are still hungry, and about 2 billion people experience moderate or severe food insecurity. The severe impact of food insecurity on malnutrition has been identified as a problem to the overall health status of people, and food security can be a contributing factor to malnutrition [48,49].

The findings from this study reveal a statistically significant difference between the experimental and control groups for knowledge (*p* < 0.001). There was a statistically significant improvement after the Triple Benefit Health Education Intervention on knowledge of participants towards malnutrition at three (*p* < 0.001) and six-months (*p* < 0.001) post-intervention compared to the baseline result. This significant improvement might be attributed to the information obtained from the Triple Benefit Health Education Intervention module. Therefore, the outcome of this study supports the effectiveness of Triple Benefit Health Education Intervention in improving the knowledge of adolescent girls. The findings of the current study regarding the effectiveness of the Triple Benefit Health Education Intervention was significantly supported by educational interventions in India (*p* < 0.05), Palestine (*p* < 0.001), Bangladesh (*p* < 0.001), Iran (*p* < 0.05), Baltimore (*p* < 0.001) and Urbana city (*p* < 0.05) [39,50,51,52,53,54,55], but contrary to a study in the U.S.A that revealed no significant difference after the intervention program (*p* = 0.45), which may be due to the absence of a theory of behavioral change, the methodological nature of the intervention program and the narrow focus of the intervention program [41]. The study by Shaaban et al. (2014) [56] further supports the view that education intervention on malnutrition is effective in increasing knowledge towards nutrition-related issues, as knowledge on nutrition-related issues is an integral achievement of healthy attitudes and practice and consequently leads to improvement of diet quality for better well-being. By using this educational intervention, knowledge significantly increased (*p* < 0.001). Similarly, the educational intervention by Shesha et al. (2018) [57] consisted of lectures, presentations, interactive discussions and the distribution of information booklets which was effective in improving the knowledge of respondents towards nutrition-related issues. The result showed significant improvement in overall knowledge (*p* < 0.05) after the educational intervention, as it has proven to bridge the gap in knowledge among adolescent girls. Knowledge is the corner stone of attitude, and limited access to knowledge has been assumed to be among the causes of malnutrition [58]. Adolescents with good nutritional knowledge have higher odds of following healthy eating habits and lifestyle, as nutrition education intervention has been promising in improving these for this age group. The use of flip chart, pictures, brainstorming, discussion, role play and group work among adolescents is less expensive, easy to use, cost effective and it holds the attention of participants since it is interactive and makes use of pictures that explain key messages which can be easily used to cater for the local needs of the people [59,60]. Improvement in nutrition related knowledge is essential in balancing the intake of food containing carbohydrate, protein, fats, mineral and vitamins. Nutritionally related dietary habits and knowledge are very significant for a healthy lifestyle, although women, children and adolescents are vulnerable because of their exceptional physiological and socioeconomic characteristics as a result of poor nutrition [61]. Adolescence is the period of growth and development in humans that occurs after childhood and before adulthood from 10 to 19 years old. Specific and unique changes occur during this phase; furthermore growth occurs in the skeleton, muscles and in every part of the body. both in systems and organs, except the brain and the head in adolescence [62]. The nutrition education intervention is a basic program to target adolescents and young children and is considered as a critical factor required to promote positive changes in individual diet, health and healthy wellbeing. Providing adolescents with nutrition related information in a multisectoral context will significantly improve adolescent knowledge towards nutrition, better dietary practice and improved health and nutritional indicators among children and adolescents [63]. Other factors such as class (SS2 *p* = 0.010) were significantly associated with good knowledge, which suggests that the triple benefit health education intervention was more effective among respondents in the higher class SS1 and SS2 compared to those in lower class JSS1, indicating that the higher the social class, the better the understanding [64]. Ethnicity (*p* < 0.025) was significantly associated with good knowledge, and socio-demographic characteristics and cultural norms and differences prevalent in the various communities could be the contributing factors to good knowledge. This study also reveals that food security (food secured; *p* = 0.001) was significantly associated with good knowledge. Food secured adolescents are more likely to have good dietary intake, contributing to healthy and good nutritional status, supporting these girls during their first pregnancy and, in turn, a positive impact on the future generation. Food insecurity or hunger reduces learning capacity, school attendance, and earning capacity, thereby increasing the risk of hunger and poverty in the future [65]. Information (good information; *p* < 0.001), motivation (good motivation; *p* < 0.001), and behavioral skills (good behavioral skills; *p* = 0.027) further reveal the significant association between knowledge of malnutrition and the IMB model, indicating that the incorporation of the IMB model in the intervention study would improve knowledge [66]. 

The results reveal a statistically significant difference in attitude between the experimental and control groups (*p* < 0.001). There was also a statistically significant difference at three (*p* < 0.001) and six-months (*p* < 0.001) post-intervention after the Triple Benefit Health Education Intervention. The findings showed that the content of the Triple Benefit Health Education Intervention module and the methodological concept had positively influenced the attitude of adolescent girls. Similarly, education intervention studies in Canada (*p* < 0.001), Palestine (*p* < 0.001), Shahr-e-kord city, Iran (*p* < 0.001), Bangladesh (*p* < 0.05), and China (*p* < 0.023) reported significant changes in post-intervention and follow up [32,42,50,51,57,67], but contrary to the interaction effect in Laram et al., 2017 [42]. In contrast, studies in Urbana (*p* > 0.05) and Bangalore (*p* > 0.05) showed no significant result in post-intervention. This may be due to a small sample size of less than 100 with less than 50 respondents in each group, inadequate training on some specific aspect of the study, presentation skills, and, more importantly, a large number of dropouts in the control group during post-intervention could be a contributing factor to its non-significance [55,57]. More so, the education intervention study showed a significant increase in attitude from baseline to post-intervention in Nigeria (*p* < 0.001) [68]. Further improvement was reported in a study in India (*p* = 0.007) from baseline to post-intervention and follow up. It appears that education interventions that concentrates on developing health-related skills and imparting health-related attitudes are more likely to benefit adolescent girls in health-enhancing practices [69]. School based nutrition intervention has proven to improve attitude among adolescents in the prevention of malnutrition. Nutrition education intervention has a long-lasting approach to building a good nutritional status among adolescents. Although the participants are from different socioeconomic contexts, the triple benefit health education intervention was effective in improving their attitude by providing valuable information as a practical solution in enhancing their attitude towards positive living [70]. Attitude can be said to be a determinant in individual choice of food and healthy living as good attitude towards malnutrition in adolescence immensely contribute to healthy lifestyle [71]. Religion (Islam; *p* = 0.001) was significantly associated with attitude. Religion has its unique way of disseminating information to its adherents and this information is transmitted through generations to meet the basic needs of its groups and for survival, shaping the attitude of individuals in the form of norms and values [72]. In terms of nutritional status (normal; *p* = 0.008), respondents with normal nutritional status significantly improved from baseline, and there was also a significant decrease in underweight from baseline as a result of the triple benefit health education intervention, giving the adolescent child hope for future survival. Food security (low food secured; *p* = 0.032) was significantly associated with attitude towards malnutrition. Food security can have impact on attitude and nutritional status thereby influencing the growth and development of adolescents as it becomes woven in an intergenerational cycle of malnutrition [73]. As for information (good information; *p* < 0.001), good information is likely to improve attitude, as acquired information on malnutrition (definition, causes, consequences and future implications) tends to change beliefs and personal values associated with various health behaviors capable of changing attitudes towards malnutrition and knowing fully the future consequences.

Findings from this study reveal a statistically significant difference for food security between the experimental and control group (*p* < 0.001). There was a statistically significant difference at three (*p* = 0.026) and six-month (*p* = 0.001) post-intervention for food security after the Triple Benefit Health Education Intervention compared to the control group at baseline. This indicated that Triple Benefit Health Education Intervention improved the food security situation of participants. The effects of malnutrition in women are borne throughout their lifecycle and through generations. Nutritional inadequacy through food insecurity during the period of adolescence can affect present and future health and well-being, as it is intrinsically linked to the health and well-being of offspring [73,74]. The interaction between food security knowledge and attitude creates a cycle whereby poor diet due to poor knowledge and attitude compromises the immune system exposing the individual to infection, poor health and wellbeing, decreased absorption of nutrients and worsened nutritional status. Most episodes of malnutrition and morbidity are linked to infection leading to wasting, stunting and underweight. The origin of wavering growth usually begins as early as during pregnancy, so there is a need for adequate nutrition and health status in the prevention of malnutrition through food security to reduce the short and long-term consequences of malnutrition. This includes the intergenerational cycle of malnutrition extended from malnourished girls becoming malnourished mothers at risk of giving birth to low birth weight infants, placing them at disadvantaged development throughout life [75]. The more knowledge and the better attitude the individuals have, the more they will prefer to acquire more information on nutritious food and be willing to buy and consume it. Knowledge of and attitude towards malnutrition influence perceptions on food security in the aspect of the quality and the quantity food and its utilization [76]. Since adolescence is a dynamic stage, diet at this stage is driven by immediate needs, and choice of food is influenced by energy needs and what will satisfy their hunger, more so because adolescents have more control, choice and responsibility regarding the household diet [77]. Eradicating food insecurity depends entirely on agricultural productivity, in line with the United Nations Millennium Development Goals to eradicate poverty and hunger, but this can be feasible in Borno state, Nigeria if the rural dwellers are back in their respective communities rather than camping in the urban areas as refugees with limited land for agricultural production, due to the prolonged insurgency threatening the peace and unity of the country [78,79,80]. Furthermore, other factors associated with food security include place of residence (urban; *p* = 0.007). This study reveals that urban residence has higher odds of being food secure compared to rural dwellers, perhaps due to non-availability of sufficient farm produce and the financial resources caused by displacement as a result of the lingering humanitarian crises that have limited farm production and food availability in the rural areas and the outskirts of the urban areas. As regards education [78,79,80,81] and monthly income (₦31,000–₦50,000; *p* = 0.012, ₦51,000 and above; *p* < 0.001), this study reveals that households with better income have higher odds of being food secured compared to lower income households. Income is an important determinant of food insecurity. Higher household income reduces the severity of food insecurity, as lack of financial security can lead to food insecurity. High household income means earning more, making more food choices and better management of the household [82,83]. As regards education of mother (primary education; *p* = 0.015, tertiary education; *p* = 0.027), mothers who are educated might have less tendency to become food insecure, as since they are educated there is tendency for them to be working or in menial business, earning income that will help in cushioning the family needs in term of food choice, availability and utilization. Mothers with higher level of education are more likely to earn income, adopt technology and be engaged in livelihood activities that can contribute to better food production, thereby reducing the risk of household food insecurity [84].

One of the strengths of this study is that it was a randomized control trial with single blinding and a validated questionnaire. The study design and its tools allowed the assessment of knowledge, attitude and food security. To the best of our knowledge, there has been no previous intervention study among adolescent girls aged 10 to 19 years on nutrition-related knowledge, attitude and food security in the northeastern part of Nigeria and no previous research available for these age groups using a randomized controlled trial. This was the first intervention module on malnutrition developed based on the IMB model. Subsequently, findings from this study can be used as fundamental data for future studies in Borno State, the northeastern part of Nigeria and Nigeria at large.

Another limitation of this study was that the intervention was conducted in only four governments secondary schools in Maiduguri Metropolitan Council (MMC). The study was conducted in only one out of the 27 districts in the state. Another limitation of the intervention includes non-inclusion of schools with married women, schools with boys only, private schools, primary schools with early adolescents, school dropouts, and adolescents not attending any school at all. Other limitations of our intervention include limited time, lack of funding, and inability to strategize for a parent teacher’s association (PTA) meeting before the onset of the intervention to enable full participation of all eligible participants.

## 5. Conclusions

Globally, humanitarian and developmental agencies have acknowledged the importance of recognizing that adolescents belong to a separate age group, from children to young adults. Adolescents, especially girls, poorly transit into adulthood at the risk of being pushed into the vicious cycle of the intergenerational cycle of malnutrition, deficiency and poverty. In this study the Triple Benefit Health Education Intervention was found to be effective in improving knowledge, attitudes and food security towards malnutrition among adolescent girls in Maiduguri Metropolitan Council, Borno State, Nigeria. Participants in the intervention group had higher odds of having good knowledge and attitude and also had higher odds of being in the food secured level at three and six-months post intervention compared to those at baseline due to the impact of the Triple Benefit Health Education Intervention. This health education intervention towards malnutrition among adolescents will go a long way in giving them a better future life. There is need for more awareness among adolescent girls for both school based and community based intervention in the future to ensure all adolescents across the community benefit from health education intervention for the betterment of their future family. 

## Figures and Tables

**Table 1:** Illustration of Triple Benefit Intervention Module using Information Motivation Behavioral Skill Model.

Module	Theory Construct	Content	Strategy	Estimated Time
Module 1	Information and behavioral skills	Definitions, forms and causes of malnutrition	Lecture, brainstorming and discussion	1 h 30 min
Module 2	Information and behavioral skills	Sign, consequences and prevention of malnutrition	Lectures, brainstorming and role play	1 h 30 min
Module 3	Information and behavioral skills	Food Groups (macro and micronutrient).	matching food	1 h 30 min
Module 4	Motivation	Prevention of malnutrition, participant’s experiences and those of other adolescent girls.	Brainstorming, discussion	1 h 30 min
Module 5	Information and behavioral skills	Intergenerational Cycle of Malnutrition, Food Groups by Food and Agriculture Organisation of the United Nations (FAO),	Lectures, discussion and kitchen/backyard garden.	1 h 30 min
Module 6	Motivation	Precautionary measures, the norms of their community, and best ways they can spread what they have learnt to their families and peers for sustainability.	Brainstorming, discussion	1 h 30 min

**Table 2:** Baseline Comparison of Sociodemographic Characteristics Between Intervention and Control Groups.

Variables	Experimental *n* (%)/Mean ± SD	Control *n* (%)/Mean ± SD	Total *n* (%)	X^2^/t	*p*-Value
Age of adolescent girls (Years)	15.0 ± 2.0	15.0 ± 2.0		−3.390 ^e^	0.697
Early adolescents (10–13)	55 (26.4)	52 (24.9)	107 (25.7)	0.171 ^d^	0.918
Middle adolescents (14–16)	101 (48.6)	102 (48.8)	203 (48.7)		
Late adolescents (17–19)	52 (25.0)	55 (26.3)	107 (25.7)		
Class ^a^				0.704 ^d^	0.983
JSS1	36 (17.3)	31 (14.8)	67 (16.1)		
JSS2	39 (18.8)	41 (19.6)	80 (19.2)		
JSS3	32 (15.4)	30 (14.4)	62 (14.9)		
SS1	29 (13.9)	30 (14.4)	59 (14.1)		
SS2	45 (21.6)	47 (22.5)	92 (22.1)		
SS3	27 (13.0)	30 (14.4)	57 (13.7)		
Ethnicity				10.494 ^d^	0.232
Bura	35 (16.8)	16 (7.7)	51 (12.2)		
Kanuri	61 (29.3)	76 (36.4)	137 (32.9)		
Hausa	13 (6.3)	13 (6.2)	26 (6.2)		
Marghi	18 (8.7)	21 (10.0)	39 (9.4)		
Shuwa	13 (6.3)	18 (8.6)	31 (7.4)		
Fulani	17 (8.2)	20 (9.6)	37 (8.9)		
Chibok	14 (6.7)	11 (5.3)	25 (6.0)		
Gwoza	20 (9.6)	18 (8.6)	38 (9.1)		
Other ethnic groups ^b^	17 (8.2)	16 (7.7)	33 (7.9)		
Religion				0.035 ^d^	0.851
Christianity	57 (27.4)	59 (28.2)	116 (27.8)		
Islam	151 (72.6)	150 (71.8)	301 (72.2)		
Place of residence				1.991 ^d^	0.158
Rural	30 (14.4)	41 (19.6)	71 (17.0)		
Urban	178 (85.6)	168 (80.4)	346 (83.0)		
Household size				0.021 ^d^	0.989
≤5 members	15 (7.2)	15 (7.2)	30 (7.2)		
6–8 members	77 (37.0)	76 (36.4)	153 (36.7)		
≥9 members	116 (55.8)	118 (56.5)	234 (56.1)		
Monthly income				1.578 ^d^	0.664
Less than ₦18,000	53 (25.5)	44 (21.1)	97 (23.3)		
₦18,000–₦30,000	65 (31.3)	69 (33.0)	134 (32.1)		
₦31,000–₦50,000	38 (18.3)	36 (17.2)	74 (17.7)		
₦51,000 and above	52 (25.0)	60 (28.7)	112 (26.9)		
Head of household				2.466 ^d^	0.291
Father	185 (88.9)	176 (84.2)	361 (86.6)		
Mother	16 (7.7)	20 (9.6)	36 (8.6)		
Relations	7 (3.4)	13 (6.2)	20 (4.8)		
Age group of the father (Years)	54.1 ± 9.6	54.6 ± 9.9		−0.534 ^e^	0.593
35 to 44	28 (13.5)	25 (12.0)	53 (12.7)	0.211 ^d^	0.646
≥45	180 (86.5)	184 (88.0)	364 (87.3)		
Education of father				7.482 ^d^	0.112
No education	25 (12.0)	25 (12.0)	50 (12.0)		
Informal education	17 (8.2)	33 (15.8)	50 (12.0)		
Primary education	6 (2.9)	8 (3.8)	14 (3.4)		
Secondary education	78 (37.5)	61 (29.2)	139 (33.3)		
Tertiary education	82 (39.4)	82 (39.2)	164 (39.3)		
Occupation of fathers				6.324 ^d^	0.097
Civil service	75 (36.1)	90 (43.1)	165 (39.6)		
Trading/business	95 (45.7)	98 (46.9)	193 (46.3)		
There Farming	21 (10.1)	12 (5.7)	33 (7.9)		
Other occupation ^c^	17 (8.2)	9 (4.3)	26 (6.2)		
Age group of the mother (Years)	41.6 ± 8.1	40.9 ± 8.3		0.850 ^e^	0.396
≤34	29 (13.9)	39 (18.7)	68 (16.3)	1.990 ^d^	0.370
35 to 44	102 (49.0)	92 (44.0)	194 (46.5)		
≥45	77 (37.0)	78 (37.3)	155 (37.2)		
Education of mothers				7.743 ^d^	0.101
No education	39 (18.8)	41 (19.6)	80 (19.2)		
Informal education	26 (12.5)	44 (21.1)	70 (16.8)		
Primary education	13 (6.3)	9 (4.3)	22 (5.3)		
Secondary education	81 (38.9)	80 (38.3)	161 (38.6)		
Tertiary education	49 (23.6)	35 (16.7)	84 (20.1)		
Occupation of mothers				0.378 ^d^	0.945
Civil service	56 (26.9)	55 (26.3)	111 (26.6)		
Trading/business	68 (32.7)	64 (30.6)	132 (31.7)		
Farming	15 (7.2)	15 (7.2)	30 (7.2)		
House wives	69 (33.2)	75 (35.9)	144 (34.9)		
Family type				0.194 ^d^	0.660
Monogamy	123 (59.1)	128 (61.2)	251 (60.2)		
Polygamy	85 (40.9)	81 (38.8)	166 (39.8)		
single parenting					

^a^ Junior secondary school (JSS), Senior secondary school (SS) ^b^ Karekare, Kilba, Minchika, Manga, Tambai, Yoruba, Mandara, Basaye, Angas, Terawa, Kanakuru, Nupe. ^c^ Malami (Voluntary Quranic teacher), ^d^ Chi-square, ^e^ t-value, SD = Standard deviation.

**Table 3:** Mean Scores for Knowledge, Attitude and Food Security at Baseline.

Variable	Experimental Mean ± SD (*n* = 208)	Control Mean ± SD (*n* = 209)	Overall Sample Mean ± SD (*n* = 417)	Minimum-Maximum	t	*p*-Value
Knowledge	8.75 ± 4.2	8.98 ± 4.3	8.87 ± 4.3	0–19	0.540	0.589
Attitude	53.28 ± 5.1	53.45 ± 4.9	53.36 ± 5.0	41–70	0.327	0.744
Food security	5.51 ± 3.4	5.47 ± 3.6	5.49 ± 3.5	0–9	−0.120	0.904

t = independent *t*-test.

**Table 4:** Comparison of Knowledge of Malnutrition between Groups and Time Points using GEE.

Variables	B	SE	Crude Odd Ratio Exp (B)	Wald Chi-Square	95% CI		*p*-Value
					Lower Bound	Upper Bound	
Groups							
Control	Ref						
Experimental	1.853	0.160	6.380	134.597	4.665	8.725	<0.001 *
Time points							
Baseline	Ref						
3-months Post-intervention	2.261	0.209	9.595	117.194	6.371	14.449	<0.001 *
6-months Post-intervention	2.708	0.211	14.993	165.012	9.919	22.662	<0.001 *

* Significant < 0.05; SE = Standard error; CI = Confidence Interval; Ref = Reference category; B = Unstandardized beta.

**Table 5:** Effectiveness of Triple Benefit Health Education Intervention on Knowledge towards Malnutrition between Groups and Time Points (baseline, 3 and 6-month post-intervention) adjusted with Covariates using GEE.

Variables	B	SE	Adjusted Odds Ratio Exp (B)	Wald Chi-Square	95% CI		*p*-Value
					Lower Bound	Upper Bound	
Intercept	−3.078	0.413					
Groups							
Control	Ref						
Experimental	0.061	0.297	1.063	0.042	0.594	1.901	0.837
Time points							
Baseline	Ref						
3-months Post-intervention	1.426	0.298	4.164	22.882	2.321	7.471	<0.001 *
6-months Post-intervention	1.759	0.303	5.805	33.656	3.204	10.515	<0.001 *
Interaction							
Control * baseline	Ref						
Experimental * 3-months Post-intervention	1.483	0.413	4.407	12.929	1.964	9.893	<0.001 *
Experimental * 6-months Post-intervention	2.238	0.446	9.379	25.212	3.915	22.471	<0.001 *
Class ^a^							
JSS1	Ref						
JSS2	0.458	0.269	1.580	2.905	0.934	2.675	0.088
JSS3	−0.023	0.295	0.977	0.006	0.548	1.743	0.937
SS1	0.586	0.296	1.797	3.928	1.007	3.208	0.047 *
SS2	0.631	0.257	1.879	6.019	1.135	3.109	0.014 *
SS3	0.454	0.285	1.575	2.537	0.901	2.755	0.111
Ethnicity							
Bura	Ref						
Kanuri	−0.021	0.252	0.979	0.007	0.597	1.605	0.932
Hausa	−0.672	0.393	0.510	2.927	0.236	1.103	0.087
Marghi	−0.080	0.343	0.923	0.054	0.471	1.809	0.816
Shuwa	−0.761	0.396	0.467	3.665	0.214	1.018	0.056
Fulani	0.351	0.367	1.420	0.913	0.691	2.918	0.339
Chibok	−0.776	0.357	0.460	4.717	0.228	0.927	0.030 *
Gwoza	−0.078	0.093	0.925	0.063	0.505	1.697	0.802
Other ethnic groups	−0.295	0.377	0.745	0.611	0.356	1.559	0.434
Food security ^a^							
Very low food secured	Ref						
Low food secured	0.246	0.197	1.279	0.632	0.870	1.881	0.210
Food secured	0.656	0.197	1.926	1.042	1.309	2.835	0.001 *
Information ^a^							
Poor information	Ref						
Good information	0.812	0.175	2.252	21.644	1.600	3.170	<0.001 *
Motivation ^a^							
Poor motivation	Ref						
Good motivation	0.680	0.184	1.973	13.688	1.377	2.829	<0.001 *
Behavioral skills ^a^							
Poor behavioral skill	Ref						
Good behavioral skill	0.383	0.173	1.466	4.902	1.045	2.057	0.027 *

* Significant < 0.05; SE = Standard error; CI = Confidence Interval; Ref = Reference category; B = Unstandardized beta; QIC = 1150.370; QICC = 1148.529, ^a^ Covariates.

**Table 6:** Comparison of Attitude towards Malnutrition between Groups and Time Points (baseline to three and six-months post-intervention) respectively using GEE.

Variables	B	SE	Crude Odd Ratio Exp (B)	Wald Chi-Square	95% CI		*p*-Value
					Lower Bound	Upper Bound	
Groups							
Control	Ref						
Experimental	0.694	0.109	2.002	40.970	1.619	2.476	<0.001 *
Time points							
Baseline	Ref						
3-months Post-intervention	0.667	0.150	1.949	19.702	1.451	2.616	<0.001 *
6-months Post-intervention	0.822	0.151	2.276	29.597	1.692	3.060	<0.001 *

* Significant < 0.05; SE = Standard error; CI = Confidence Interval; Ref = Reference category; B = Unstandardized beta.

**Table 7:** Effectiveness of Triple Benefit Health Education Intervention on Attitude towards Malnutrition between Groups and Time Points (baseline, three and six post-intervention) adjusted with Covariates using GEE.

Variables	B	SE	Crude Odd Ratio Exp (B)	Wald Chi-Square	95% CI		*p*-Value
					Lower Bound	Upper Bound	
Intercept	−1.237	0.210					
Groups							
Control	Ref						
Experimental	0.000	0.201	1.000	0.000	0.674	1.482	0.998
Time points							
Baseline	Ref						
3-months Post-intervention	0.248	0.205	1.624	5.596	1.087	2.428	0.018 *
6-months Post-intervention	0.485	0.216	1.282	1.324	0.840	1.957	0.250
Interaction							
Control * baseline	Ref						
Experimental * 3-months Post-intervention	1.124	0.308	1.367	5.027	1.747	2.501	0.017 *
Experimental * 6-months Post-intervention	0.312	0.322	3.076	12.160	1.636	5.785	<0.001 *
Religion ^a^							
Christianity	Ref						
Islam	0.409	0.120	1.505	1.027	1.189	1.903	0.001 *
Nutritional status (BMI percentile) ^a^							
Underweight	Ref						
Normal	0.349	0.148	1.480	7.012	1.107	1.978	0.008 *
Overweight	0.214	0.494	1.238	0.187	0.470	3.263	0.666
Obese	0.392	0.148	1.418	0.213	0.321	6.256	0.645
Food security ^a^							
Very low food secured	Ref						
Low food secured	0.346	0.161	1.414	4.622	1.031	1.939	0.032 *
Food secured	0.208	0.144	1.231	2.089	0.929	1.632	0.148
Information ^a^							
Poor information	Ref						
Good information	0.498	0.131	1.645	14.344	1.271	2.128	<0.001 *

* Significant < 0.05; SE = Standard error; CI = Confidence Interval; Ref = Reference category; B = Unstandardized beta; BMI = Body mass index, QIC = 1611.735; QICC = 1612.245, ^a^ Covariates.

**Table 8:** Comparison of Food Security towards Malnutrition between Groups and Time Points (baseline to three and six-months) respectively using GEE.

Variables	B	SE	Crude Odd Ratio Exp (B)	Wald Chi-Square	95% CI		*p*-Value
					Lower Bound	Upper Bound	
Groups							
Control	Ref						
Experimental	1.545	0.127	4.688	147.643	3.654	6.015	<0.001 *
Time points							
Baseline	Ref						
3-months Post-intervention	0.303	0.137	1.356	4.968	1.037	1.771	0.026 *
6-months Post-intervention	0.463	0.134	1.589	12.019	1.223	2.064	0.001 *

* Significant < 0.05; SE = Standard error; CI = Confidence Interval; Ref = Reference category; B = Unstandardized beta.

**Table 9:** Effectiveness of Triple Benefit Health Education Intervention on Food Security towards Malnutrition between Groups (intervention and control) and Time Points (baseline, 3 and 6-months post-intervention) adjusted with Covariates using GEE.

Variables	B	SE	Crude Odd Ratio Exp (B)	Wald Chi-Square	95% CI		*p*-Value
					Lower Bound	Upper Bound	
Groups							
Control	Ref						
Experimental	−0.120	0.195	0.887	0.378	0.606	1.299	0.539
Time points							
Baseline	Ref						
3-months Post-intervention	−0.089	0.202	0.914	0.472	0.616	1.358	0.658
6-months Post-intervention	−0.141	0.205	0.869	0.196	0.581	1.299	0.492
Interaction							
Control * baseline	Ref						
Experimental * 3-months Post-intervention	0.749	0.291	2.116	6.651	1.197	3.740	0.010
Experimental * 6-months Post-intervention	0.937	0.283	2.552	10.995	1.467	4.440	0.001
Place of residence ^a^							
Rural	Ref						
Urban	0.427	0.159	1.532	7.220	1.122	2.091	0.007 *
Monthly income ^a^							
Less than ₦18,000	Ref						
₦18,000–₦30,000	0.027	0.158	1.311	2.950	0.963	1.785	0.086
₦31,000–₦50,000	0.431	0.172	1.539	6.309	1.099	2.153	0.012 *
₦51,000 and above	0.805	0.154	2.236	27.171	1.652	3.025	<0.001 *
Education of mothers ^a^							
No education	Ref						
Informal education	0.023	0.183	1.023	0.016	0.715	1.646	0.900
Primary education	0.553	0.227	1.739	5.946	1.115	2.712	0.015 *
Secondary education	0.150	0.152	1.162	0.983	0.863	1.564	0.321
Tertiary education	0.374	0.169	1.454	4.915	1.044	2.025	0.027 *
Source of information ^a^							
Mass/social media	Ref						
Family/friends	−0.287	0.245	1.361	1.583	0.842	2.200	0.208
Health worker/clinic	−0.495	0.271	0.507	6.294	0.298	0.862	0.012 *
School teacher	−0.679	0.242	0.610	4.181	0.379	0.980	0.041 *
Health education program	0.308	0.258	0.750	1.237	0.452	1.245	0.266
Protein ^a^	0.003	0.001	1.003	4.605	1.000	1.005	0.032 *

* Significant < 0.05; SE = Standard error; CI = Confidence Interval; Ref = Reference category; B = unstandardized beta; ^a^ Covariates.

## Data Availability

Not applicable.

## References

[1] Ashraf Z., Hussain M., Majeed I., Afzal M., Parveen K.S.A.G. (2019). Effectiveness of Health Education on Knowledge and Practice Regarding the Importance of Well-Balanced Nutrition Among School Students. J. Health Med. Nurs..

[2] Sharma M., Watode B., Srivastava A. (2017). Nutritional Status of Primary School Children through Anthropometric Assessment in Rural Areas of Moradabad. Ann. Int. Med. Dent. Res..

[3] Shariff Z.M., Bukhari S.S., Othman N., Hashim N., Ismail M., Kasim S.M., Paim L., Samah B.A., Azhar Z., Hussein M. (2008). Nutrition Education Intervention Improves Nutrition Knowledge, Attitude and Practices of Primary School Children: A Pilot Study. Int. Electron. J. Health Educ..

[4] Pushpa K.S., Rani D.J. (2015). Nutritional profile of children (0–5 years) in the service villages of Gandhigram rural institute. Curr. Res. Nutr. Food Sci..

[5] Yeganeh S., Motamed N., Najafpourboushehri S., Ravanipour M. (2018). Assessment of the knowledge and attitude of infants’ mothers from Bushehr (Iran) on food security using anthropometric indicators in 2016: A cross-sectional study. BMC Public Health.

[6] World Health Organization (2018). Guidelines: Implementing Effective Actions for Improving Adolescents Nutrition.

[7] HLPE (2017). Nutrition and Food Systems. A Report by the High Level Panel of Experts on Food Security and Nutrition of the Committee on World Food Security.

[8] FAO, WHO, UNICEF (2017). Sustainable Food Systems for Healthy Diets in Europe and Central Asia a Joint FAO/WHO Regional Symposium and Initiative in Collaboration of UNICEF and WFP.

[9] FAO (2016). Influencing Food Environments for Healthy Diets.

[10] United Nations Children’s Fund (UNICEF) (2019). The State of the World’s Children 2019: Children, Food and Nutrition. Growing Well in a Changing World.

[11] Caldeira S., Storcksdieckgennant B., Bakogianni I., Gauci C., Calleja A., Furtado A. (2017). Public Procurement of Food for Health: Technical Report on School Setting.

[12] Hyska J., Burazeri G., Menza V., Dupouy E. (2020). Assessing nutritional status and nutrition-related knowledge, attitudes and practices of Albanian schoolchildren to support school food and nutrition policies and programmes. Food Policy.

[13] Bundy D., de Silva N., Horton S., Jamison D., Patton G. (2018). Re-Imagining School Feeding: A High-Return Investment in Human Capital and Local Economies.

[14] Bundy D., de Silva N., Horton S., Jamison D., Patton G. (2018). Optimizing Education Outcomes: High-Return Investments in School Health for Increased Participation and Learning.

[15] Coppoolse H.L., Seidell J.C., Dijkstra S.C. (2020). Impact of nutrition education on nutritional knowledge and intentions towards nutritional counselling in Dutch medical students: An intervention study. BMJ Open.

[16] Kıvrak A.O., Altın M. (2018). Nutrition Knowledge and Attitude Change of Students Studying in State and Private Secondary Schools. J. Educ. Train. Stud..

[17] Rajikan R., Esmail S. (2020). Nutrition Education to Improve Nutrition Knowledge, Attitude and Practice among Yemeni School Children. Trends Appl. Sci. Res..

[18] National Bureau of Statistics (2020). 2019 Poverty and Inequality in Nigeria: Executive Summary.

[19] Owoo N.S. (2020). Demographic considerations and food security in Nigeria. J. Soc. Econ. Dev..

[20] Nwozor A., Olanrewaju J.S., Ake M.B. (2019). National insecurity and the challenges of food security in Nigeria. Acad. J. Interdiscip. Stud..

[21] The Federal Republic of Nigeria (2016). The Federal Republic of Nigeria Agricultural Sector Food Security and Nutrition Strategy 2016–2025.

[22] National Population Commission (NPC), ICF (2019). Nigeria Demographic Health Survey 2018.

[23] National Beareu of Statistics (NBS) (2018). National Nutrition and Health Survey.

[24] Parul C., Smith R.E. (2018). Adolescent Undernutrition: Global Burden, Physiology, and Nutritional Risks. Ann. Nutr. Metab..

[25] Ministry of Budget and National Planning (2016). National Policy on Food and Nutrition in Nigeria.

[26] International Food Policy Research Institute (IFPRI) (2019). Nutrition Policy in Nigeria.

[27] Federal Ministry of Health (2005). National Plan of Action on Food and Nutrition in Nigeria.

[28] Adinma J.I.B., Adinma A.E., Umeononihu O.S. (2017). Nutrition Policy and Practice Landscape on Adolescent, Pre-Pregnancy and Maternal Nutrition in Nigeria. JOJ Nurs. Health Care.

[29] Save the Children (2016). Scaling Up Nutrition Interventions for Children Left Behind in Nigeria.

[30] Peyman N., Abdollahi M. (2016). Using of Information-Motivation-Behavioral Skills Model on Nutritional Behaviors in Controlling Anemia among Girl Students. J. Res. Health.

[31] Lemeshow S., Hosmer D.W., Klar J., Lwanga S.K. (1990). Adequacy of Sample Size in Health Studies.

[32] Wang D., Stewart D., Chang C., Shi Y. (2015). Effect of a school-based nutrition education program on adolescents’ nutrition-related knowledge, attitudes and behaviour in rural areas of China. Environ. Health Prev. Med..

[33] Macías Y.F., Glasauer P. (2014). Guidelines for Assessing Nutrition-Related Knowledge, Attitudes and Practices Manual.

[34] Govender D., Naidoo S., Taylor M. (2019). Knowledge, Attitudes and Peer Influences related to Pregnancy, Sexual and Reproductive Health among Adolescents using Maternal Health Services in Ugu, KwaZulu-Natal, South Africa. BMC Public Health.

[35] Chanyalew W., Kassahun A.G.M. (2017). Knowledge, Attitude, Practices and their Associated Factors towards Diabetes Mellitus among non Diabetes Community members of Bale Zone Administrative Towns, South East. PLoS ONE.

[36] Carol L.C., Nord M., Kristi L.L., Yadrick K. (2006). Self-Administered Food Security Survey Module for Children Ages 12 Years and Older. J. Nutr..

[37] Shtasel-Gottlieb Z., Palakshappa D., Yang F., Goodman E. (2016). The Relationship Between Developmental Assets and Food Security in Adolescents from a Low-Income Community. J. Adolesc. Health.

[38] Dziura J.D., Post L.A., Zhao Q., Fu Z., Peduzzi P. (2013). Strategies for Dealing with Missing Data in Clinical Trials: From Design to Analysis. Yale J. Biol. Med..

[39] Shin A., Surkan P.J., Coutinho A.J., Suratkar S.R., Campbell R.K., Rowan M., Sharma S., Dennisuk L.A., Karlsen M., Gass A. (2015). Impact of Baltimore Healthy Eating Zones: An Environmental Intervention to Improve Diet Among African American Youth. Health Educ. Behav..

[40] Zhou W., Xu X., Li G., Sharma M., Qie Y., Zhao Y. (2016). Effectiveness of a school-based nutrition and food safety education program among primary and junior high school students in Chongqing, China. Glob. Health Promot..

[41] Robert G., LaChausse A. (2017). Clustered Randomized Controlled Trial to Determine Impacts of the Harvest of the Month Program. Health Educ. Res..

[42] Laramee C., Drapeau V., Valois P., Goulet C., Jacob R., Provencher V., Lamarche B. (2017). Evaluation of a Theory-Based Intervention Aimed at Reducing Intention to Use Restrictive Dietary Behaviors among Adolescent Female Athletes. J. Nutr. Educ. Behav..

[43] Karimi-Shahanjarini A., Rashidian A., Omidvar N., Majdzadeh R. (2013). Assessing and Comparing the Short-Term Effects of TPB Only and TPB Plus Implementation Intentions Interventions on Snacking Behavior in Iranian Adolescent Girls: A Cluster Randomized Trial. Am. J. Health Promot..

[44] Tamiru D., Argaw A., Gerbaba M., Ayana G., Nigussie A., Belachew T. (2016). Effect of integrated school-based nutrition education on optimal dietary practices and nutritional status of school adolescents in Southwest of Ethiopia: A quasi-experimental study. Int. J. Adolesc. Med. Health.

[45] Mokhtari F., Kazemi A., Ehsanpour S. (2017). Effect of educational intervention program for parents on adolescents’ nutritional behaviors in Isfahan in 2016. J. Educ. Health Promot..

[46] Bogart L.M., Cowgill B.O., Elliott M.N., Klein D.J., Hawes-Dawson J., Uyeda K., Elijah J., Binkle D.G., Schuster M.A. (2014). A Randomized Controlled Trial of Students for Nutrition and EXercise: A Community-Based Participatory Research Study. J. Adolesc. Health.

[47] Kupolati M.D., Macintyre U.E., Gericke G.J., Becker P. (2019). A Contextual Nutrition Education Program Improves Nutrition Knowledge and Attitudes of South African Teachers and Learners. Front. Public Health.

[48] Food and Agriculture Organization of the United Nations (2008). An Introduction to the Basic Concepts of Food Security.

[49] FAO, IFAD, UNICEF (2019). The State of Food Security and Nutrition in the World 2019. Safeguarding against Economic Slowdowns and Downturns.

[50] Razzak M.A., Al Hasan S.M., Rahman S.S., Asaduzzaman M. (2016). Role of nutrition education in improving the nutritional status of adolescent girls in North West areas of Bangladesh. Int. J. Sci. Eng. Res..

[51] Jalambo M.O., Sharif R., Naser I.A., Karim N.A. (2017). Improvement in Knowledge, Attitude and Practice of Iron Deficiency Anaemia among Iron-Deficient Female Adolescents after Nutritional Educational Intervention. Glob. J. Health Sci..

[52] Saraf D.S., Gupta S.K., Pandav C.S. (2014). Effectiveness of a School Based Intervention for Prevention of Non-communicable Diseases in Middle School Children of Rural North India: A Randomized Controlled Trial. Indian J. Pediatr..

[53] Bandyopadhyay L., Maiti M., Dasgupta A., Paul B. (2017). Intervention for Improvement of Knowledge on Anemia Prevention: A School-based Study in a Rural area of West Bengal. Int. J. Health Allied Sci..

[54] Kaveh M.H., Darabi F., Khalajabadi-Farahani F., Yaseri M., Kaveh M.H., Mohammadi M.J., Reza H., Behrooz A., Shojaeizadeh A.R.D. (2018). The Impact of a TPB-Based Educational Intervention on Nutritional Behaviors in Iranian Adolescent Girls: A Randomized Controlled Trial. Fresenius Environ. Bull..

[55] Oakley A.R., Nelson S.A., Nickols-Richardson S.M. (2017). Peer-Led Culinary Skills Intervention for Adolescents: Pilot Study of the Impact on Knowledge, Attitude, and Self-efficacy. J. Nutr. Educ. Behav..

[56] Shaaban S.Y., Nassar M.F., Shatla R.H., Deifallah S.M. (2014). Nutritional Knowledge, Attitude and Practice of Predominantly Female Preschool Teachers: Effect of Educational Intervention. Br. J. Med. Med. Res..

[57] Shesha T., Chaluvaraj I., Satyanarayana P.T. (2018). Change in Knowledge, Attitude and Practice Regarding Anaemia among High School Girls in Rural Bangalore: An Health Educational Interventional Study. Natl. J. Community Med..

[58] Agustina R., Wirawan F., Sadariskar A.A., Ked S., Setianingsing A.A., Nadiya K., Prafiantini E., Asri E.K., Purwanti T.S., Kusyuniati S. (2021). Associations of Knowledge, Attitude, and Practices toward Anemia with Anemia Prevalence and Height-for-Age Z-Score among Indonesian Adolescent Girls. Food Nutr. Bull..

[59] Kameshwary R., Thakur A., Mangal A., Vaghela J.F., Banerjee S., Gupta V. (2020). A study to assess the effectiveness of a nutrition education session using flipchart among school-going adolescent girls. J. Educ. Health Promot..

[60] Bharti R., Marwaha A., Badshah T., Sengupta R. (2021). Effectiveness of a Nutritional Education Intervention Focussed on Iron among School Children in National Capital Region and Mumbai. J. Clin. Diagn. Res..

[61] World Health Organization (2006). Adolescent Nutrition: A Review of the Situation in Selected South-East Asian Countries.

[62] Bhuiyan F.R., Barua J.L., Kalam K.A. (2021). Knowledge, Attitude and Practices Regarding Nutrition among Adolescent Girls in Dhaka City: A Cross-sectional Study. Nutr. Food Sci. Int. J..

[63] Hewett P.C., Willig A.L., Digitale J., Soler-Hampejsek E., Behrman J.R., Austrian K. (2020). Assessment of an adolescent-girl-focused nutritional educational intervention within a girls’ empowerment programme: A cluster randomised evaluation in Zambia. Public Health Nutr..

[64] Viggiano A., Viggiano E., di Costanzo A., Viggiano A., Scianni G., Santangelo C., Battista R., Monda M., Viggiano A., de Luca B. (2015). Kaledo, a board game for nutrition education of children and adolescents at school: Cluster randomized controlled trial of healthy lifestyle promotion. Eur. J. Pediatr..

[65] FAO (2005). The State of Food Insecurity in the World: Eradicating World Hunger—Key to Achieving the Millennium Development Goals.

[66] Shapu R.C., Ismail S., Ahmad N., Ying L.P., Abubakar I. (2020). Knowledge, Attitude, and Practice of Adolescent Girls Towards Reducing Malnutrition in Maiduguri Metropolitan Council, Borno State, Nigeria: Cross-Sectional Study. Nutrients.

[67] Vardanjani A.E., Reisi M., Javadzade H., Pour Z.G. (2015). The Effect of Nutrition Education on Knowledge, Attitude, and Performance about Junk Food Consumption among Students of Female Primary Schools. J. Educ. Health Promot..

[68] Ogunsile S.E., Ogundele B.O. (2016). Effect of game-enhanced nutrition education on knowledge, attitude and practice of healthy eating among adolescents in Ibadan, Nigeria. Int. J. Health Promot. Educ..

[69] Anand T., Ingle G.K., Meena G.S., Kishore J., Yadav S. (2015). Effect of Life Skills Training on Dietary Behavior of School Adolescents in Delhi: A Nonrandomized Interventional Study. Asia-Pac. J. Public Health.

[70] Abu-Baker N.N., Eyadat A.M., Khamaiseh A.M. (2021). The impact of nutrition education on knowledge, attitude, and practice regarding iron deficiency anemia among female adolescent students in Jordan. Heliyon.

[71] Aduroja A.E., Remo I. (2021). Dietary Knowledge and Attitudes of In-School Adolescents in Private Secondary Schools in Ifako-Ijaye Local Government, Lagos, Nigeria. Int. J. Innov. Sci. Res. Technol..

[72] Alonso E.B. (2015). The Impact of Culture, Religion and Traditional Knowledge on Food and Nutrition Security in Developing Countries.

[73] Das J.K., Lassi Z.S., Hoodbhoy Z., Salam R.A. (2018). Nutrition for the Next Generation: Older Children and Adolescents. Ann. Nutr. Metab..

[74] United Nations Children’s Fund (UNICEF) (2018). Food Systems for Children and Adolescents: Working Together to Secure Nutritious Diets.

[75] Ghattas H. (2014). Food Security and Nutrition in the Context of the Global Nutrition Transition.

[76] Weerasekara P.C., Withanachchi C.R., Ginigaddara G.A.S., Ploeger A. (2020). Food and Nutrition-Related Knowledge, Attitudes, and Practices among Reproductive-age Women in Marginalized Areas in Sri Lanka. Int. J. Environ. Res. Public Health.

[77] Food and Agriculture Organization of the United Nations (FAO) (2019). Strengthening Sector Policies for Better Food Security and Nutrition Results.

[78] Otaha J.I. (2013). Food Insecurity in Nigeria: Way Forward. An. Int. Multidiscip. J. Ethiop..

[79] Shapu R.C., Ismail S., Ahmad N., Lim P.Y., Njodi I.A. (2020). Food Security and Hygiene Practice among Adolescent Girls in Maiduguri Metropolitan Council, Borno State, Nigeria. Foods.

[80] Global Network Against Food Crisis (2020). 2020 Global Report on Food Crises.

[81] WFP (2018). Global Report on Food Crisis 2018.

[82] Roncarolo F., Potvin L. (2017). Associations between the local food environment and the severity of food insecurity among new families using community food security interventions in Montreal. Can. J. Public Health.

[83] Ukegbu P., Nwofia B., Ndudiri U., Uwakwe N. (2019). Food Insecurity and Associated Factors Among University Students. Food Nutr. Bull..

[84] Masa R., Chowa G., Nyirenda V. (2017). Prevalence and Predictors of Food Insecurity among People Living with HIV Enrolled in Antiretroviral Therapy and Livelihood Programs in Two Rural Zambian Hospitals. Ecol. Food Nutr..

